# A novel *badnavirus* discovered from *Betula sp*. affected by *birch leaf-roll disease*

**DOI:** 10.1371/journal.pone.0193888

**Published:** 2018-03-01

**Authors:** Artemis Rumbou, Thierry Candresse, Armelle Marais, Sebastien Theil, Juliane Langer, Risto Jalkanen, Carmen Büttner

**Affiliations:** 1 Division Phytomedicine, Albrecht Daniel Thaer-Institute, Faculty of Life Sciences, Humboldt-Universität zu Berlin, Berlin, Germany; 2 Equipe Virologie, UMR 1332 BFP, French National Institute for Agricultural Research (INRA), Villenave d'Ornon Cedex, France; 3 Rovaniemi Unit, Finnish Forest Research Institute (Metla), Natural Resources Institute, Luke, Rovaniemi, Finland; University of Basel, SWITZERLAND

## Abstract

In declining birches (*Betula* sp.) from different European stands affected by the “birch leaf-roll disease” (BLRD) a novel virus is identified by means of RNA-Seq virome analysis. The virus represents a new member in the genus Badnavirus, family *Caulimoviridae*, tentatively named *Birch leaf roll-associated virus* (BLRaV) and it is the first badnavirus found to infect birch. Complete genome sequences (7,862–7,864 nucleotides) of three viral isolates of Finnish and German origin have been determined. The virus sequences show a typical badnavirus organization with three major open reading frames (ORFs) and a fourth potential ORF overlapping with the end of ORF3. ORFs 1-2-3 show low level of amino acid identity to the corresponding proteins encoded by other badnaviruses, reaching a maximum of 44% identity (ORF3). *Grapevine vein-clearing virus* appears as the closest badnavirus when considering the polymerase region. So far, we can exclude evidence for presence of endogenous BLRaV elements in the birch genome, while evidence for the episomal activity of BLRaV is provided. The viral population holds significant haplotype diversity, while co-infection by different BLRaV variants are observed in single hosts. BLRaV presence is associated with the BLRD in both silver (*B*. *pendula)* and downy birch (*B*. *pubescens*). These results challenge the earlier hypothesis of a causal role of *Cherry leaf roll virus* in BLRD. Further work is now needed to finally prove that BLRaV is the causal agent for the BLRD.

## Introduction

With more and more results accumulating, it is now clear that virus-associated birch epidemics currently constitute a serious problem for the health of birch forest and urban stands throughout Europe [1–5]. For forestry, birch is the most important broadleaved tree species in Northern and Eastern Europe. It plays an essential role in the biodiversity of coniferous forests as a large number of species feed on or live together with birch, including mycorrhiza-forming fungi, herbivores, wood-decaying fungi and saproxylic insects [6]. Moreover, both silver and downy birch have a wide natural distribution on the Eurasian continent, ranging from the Atlantic to eastern Siberia [7]. First observations of declining birches in a wide range of environments were made in Fennoscandia, where, since the summer of 2002, increasing numbers of birch trees of various sizes and ages were discovered with foliar disorders of unknown etiology [8]. The disease was first recognised as a phytopathological problem in Finland and was described as the “birch leaf-roll disease” (BLRD) by Jalkanen et al. [8]. Diseased trees exhibit foliar symptoms including leaf rolling, vein banding, chlorosis with subsequent necrosis, gradually leading trees to decline. Symptomatic trees of different *Betula* species were reported throughout Finland, in the subarctic zone of Norway as well as in Sweden, in urban parks and forests as well as exactly at the birch alpine tree line. Similar symptoms had been previously sporadically reported in Great Britain [9, 10] and in declining birch forests in Germany [11–14]. The same symptoms were also reported recently in native mountain birch stands in Corsica [3].

Viral detection efforts in symptomatic birches originating from Finland initially targeted the nepovirus, *Cherry leaf roll virus* (CLRV), as the leaf symptoms resembled those observed in CLRV-infected birches from Germany [1, 12, 13]. CLRV could be irregularly detected in diseased *Betula* trees of various species by applying conventional molecular methods like IC-RT-PCR [8] or nested RT-PCR [15]. Further investigation of the disease showed that a birch CLRV population from Rovaniemi, Finland detected by means of nested RT-PCR holds a high degree of genetic variability, mixed infections by CLRV variants from different phylogenetic groups being present in single trees, with evidence for recombination between variants [5]. Grafting of twigs from CLRV-positive *B*. *pubescens* donor trees from Rovaniemi, Finland to non-symptomatic *B*. *pubescens* rootstocks successfully transmitted CLRV [5]. However, when testing a larger number of samples from Finland for CLRV presence, a direct correlation between CLRV presence and BLRD symptoms could not be achieved.

To investigate the hypothesis that other viruses might be involved in the etiology of this emerging disease, an RNA-Seq metagenomic analysis is performed and the results obtained are reported here. The use of high-throughput sequencing coupled to bioinformatics analysis constitutes a novel approach that has remarkably changed our ability to detect known or novel viruses without the need for any prior knowledge [16–19]. The results of the analysis of RNA-Seq data obtained using leaf tissues from graft-inoculated birch seedlings as well as from the original donor trees confirmed that the BLRD-affected *B*. *pubescens* seedlings are infected by a novel badnavirus. Badnaviruses are double-stranded DNA pararetroviruses and are one of the eight genera of the family *Caulimoviridae*. They have bacilliform particles and replicate through an RNA intermediate [20]. They are known to infect a broad range of economically important crops worldwide, causing in some of them important economic losses [21]. In the current study, the complete genome sequence of three isolates of this new virus, two from Rovaniemi, Finland and one from Berlin, Germany are reported. Partial sequences from the ORF3-coding region of other isolates obtained from symptomatic birches in Berlin and Rovaniemi are also obtained, providing information on the diversity of this novel agent.

## Materials and methods

### Plant samples

**Fig 1:**
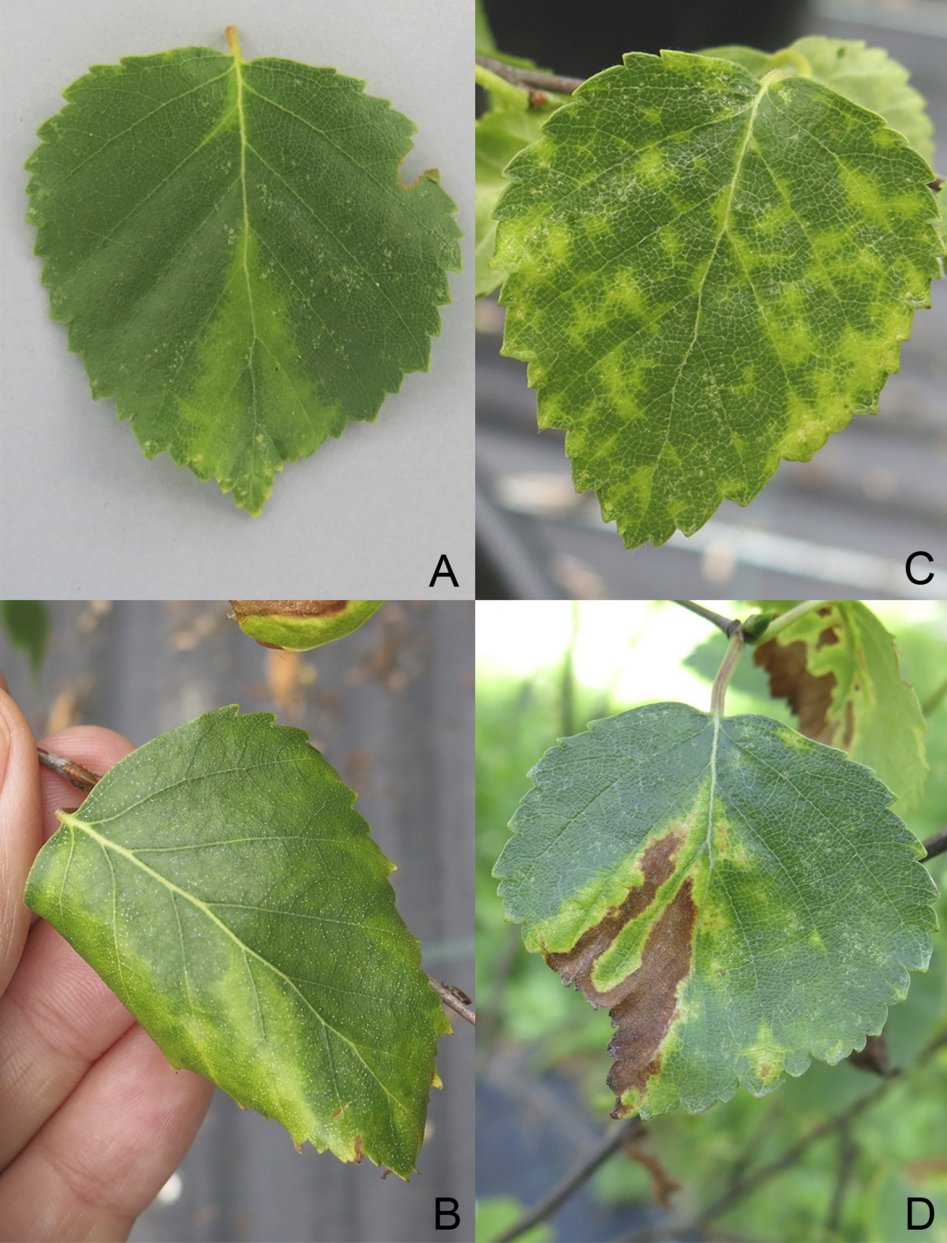
Leaf symptoms exhibited on the grafted birches infected by BLRaV. Chlorotic vein banding (A), leaf roll (B), mottling (C) and leaf necrosis (D) (later infection stage).

Virus transmission was carried out by grafting in 2011. Two twigs originating from a *B*. *pubescens* donor tree (Bpub3) with severe leaf symptoms (vein banding, leaf chlorosis and necrosis, leaf rolling) from Rovaniemi, Finland were grafted on two non-symptomatic *B*. *pubescens* rootstocks (grafted seedlings BpubFin407501_3A and BpubFin407507_3I). In parallel, one twig originating from a symptomatic, CLRV-positive *B*. *pendula* tree (Bpen 5) from Berlin, Germany was grafted on a non-symptomatic *B*. *pendula* rootstock (grafted seedling BpenGer407526_5M). The following growing season, in May 2012, symptoms similar to the ones exhibited by the donor trees could be observed on the grafted birches (Fig 1). Two birch seedlings, a CLRV-negative *B*. *pubescens* seedling (BpubGerNo4) and a CLRV-negative *B*. *pendula* seedling (BpenGerM0197542), obtained from the same nursery and not exhibiting any symptoms were used as negative controls. For the graftings and for the controls, two-year-old sprouting birch rootstocks were used (nursery Reinke GbR Baumschulen, Rellingen, Germany).

### Total RNA extractions and RNA-Seq analysis

Pooled samples of three to five leaves randomly selected from the canopy were used for RNA extractions from the symptomatic or from the asymptomatic control seedlings. Total RNAs were isolated using the InviTrap^®^ Spin Plant RNA Mini Kit (STRATEC Molecular, Germany), followed by removal of remaining DNA with rDNase according to the supplier protocol (Macherey-Nagel, Germany) and RNA purification using NucleoSpin^®^ RNA Clean-up (Macherey-Nagel, Germany). Ribosomal RNA depletion was performed using the RiboMinus Plant Kit for RNA-Seq (Invitrogen). One to two micrograms of RiboMinus RNA of each sample were used for cDNA synthesis with the Maxima H Minus double-stranded cDNA synthesis Kit (Thermo Scientific) primed with random hexamers. One to two micrograms purified double-stranded cDNA from each sample were sent to BaseClear (Netherlands) for RNA-Seq analysis with the Illumina HiSeq2500 system and 100 bp-long paired-end sequence reads corresponding to 50–100 Mb data/sample were generated.

### Sequence assembly

All NGS data processing and analysis were performed using CLC Genomics Workbench version 7.0.4. Reads were first submitted to quality filtering and trimming. The resulting cleaned reads (approx. 545,000–803,000 reads per sample) were then assembled into contigs, generating between 263 and 1,055 contigs longer than the 300 nucleotide cut-off (Table 1). Contigs were then annotated by BLASTN and BLASTX against the GenBank database. When needed, contigs representing the same badnavirus agent were manually assembled into larger scaffolds and scaffolds polished by re-mapping of reads on the scaffolds using CLC Genomics Workbench.

**Table 1:** List of samples used for RNA-Seq analysis including NGS data generated for the new badnavirus and for *Cherry leaf roll virus*.

Plant	Species	Geographic origin^a^	Symptoms^b^	Cleaned reads	Total contigs	Badnavirus contigs		CLRV contigs
						Number (length)	Average coverage	
**BpubFin407501_3A**	*B*. *pubescens*	FIN / DE	**+**	803,120	1055	7 (1,0951nt)^c^	35.1X—2.5X^d^	0
**BpubFin407507_3I**	*B*. *pubescens*	FIN / DE	**+**	613,923	838	1 (7,973nt)	68.7X	0
**BpenGer407526_5M**	*B*. *pendula*	DE / DE	**+**	725,231	769	2 (7,522nt)^c^	43.5X	10 (14,467nt)^c^
**BpubGerNo4**	*B*. *pubescens*	DE (seedling)	-	682,408	320	0		0
**BpenGerM0197542**	*B*. *pendula*	DE / DE	-	546,722	263	0		0

^a:^ for grafted plants the origin of the scion and rootstock are indicated. FIN: Finland; DE: Germany.

^b:^ a plus sign indicates plants showing symptoms of the “birch leaf-roll disease”; a minus, indicates asymptomatic plants

^c:^ when several contigs are obtained, their cumulated length is shown

^d:^ average coverage is provided separately for the two viral isolates co-infecting the plant

### Confirmation of the circular genome with PCR and variant analysis with RT-PCR

A pair of specific, outward-facing primers (PolyBadna-F1/R1; Table 2) was designed in conserved regions at the 5’ and 3’ ends of the assembled badnavirus scaffolds in order to complete the genome sequences and confirm genome circularity. PCR was performed in a 50 μl volume containing 10 mM Tris-HCl (pH 8.5), 2 mM MgCl_2_, 50 mM KCl, 0.2 mM dNTPs, PolyBadna-F1/R1 primers at 1 μM each, and 1 U of Advantage 2 Polymerase Mix (Clontech). After an initial denaturation step of 1 min at 98°C, 40 cycles were applied, each of 95°C for 30 sec, 60°C for 30 sec, and 68°C for 1 min, followed by a final extension step of 10 min at 68°C.

**Table 2:** Primers used for genome circularity verification and for specific detection of the BLRD-associated badnavirus.

Primer name	Primer sequence (5’– 3’)	Annealing temperature	Product length (bp)
**PolyBadna-F1**	ACGGCATTCAGAGAGCTACGC	60 °C	780
**PolyBadna-R1**	AATATTATTGCCAAAACACACCAAAC		
**Badna ORF3 for**	GATGGGATTCTCTGGATGAAAGC	53 °C	283
**Badna ORF3 rev**	CCCTAAAAAGGAAGCGAAAGGAT		

Pooled samples of 3 to 5 leaves from different twigs of each tree were used to confirm the presence of the new badnavirus in birches from Berlin and Rovaniemi. Primers were designed using OligoCalc [22] targeting short regions of ORF3 conserved among the different viral scaffolds (Badna ORF3 for/rev; Table 2). The first strand cDNAs were synthesized from 1 μg total RNA in a 20 μl reaction volume of 1X RT buffer (Thermo Scientific) containing 1 μM dNTPs mix, 200 U RevertAid Premium reverse transcriptase (Thermo Scientific), 20 U Ribolock RNase inhibitor (Thermo Scientific) and 100 pmol of random hexamer-oligonucleotides (Biomers.net GmbH). Subsequent PCR was conducted in a 25 μl volume of 1X DreamTaq Buffer (Thermo Scientific) containing 0.2 μM dNTP mix, 0.625 U of DreamTaq DNA polymerase and 1 μM of each forward and reverse primer (Table 2). The thermal cycles were as follows: 2 min at 95°C followed by 38 cycles at 95°C for 30 s, 53°C for 30 s, 72°C for 40 s and finished by 72°C for 5 min. Omitting the primers sequences, the amplified fragment was 241 nucleotides long. Unless specified otherwise, uncloned PCR products were directly submitted for sanger sequencing.

When needed, PCR products were first cloned prior to sequencing, in order to identify haplotype-specific rather than consensus sequences. Following purification with the MSB^®^SpinPCRapace Kit (Stratec), PCR products were ligated into the pGEM-T Easy cloning vector using the pGEM-T Easy vector system I (Promega). Transformation of JM109 competent cells was conducted following standard procedures (Promega).

### Sequence analysis and phylogenetic reconstructions

Multiple nucleotide or amino acid sequence alignments were performed using CLUSTALW in Bioedit 7.4 (Hall, 1999), as well as pairwise sequence identity calculations. For the phylogenetic comparisons of complete ORF3 sequences, a representative sequence was used for each badnavirus. *Rice tungro bacilliform virus*, the type member of the genus *Tungrovirus* (family *Caulimoviridae*), was used as outgroup. Bootstrapped Maximum likelihood (ML) and Neighbour-Joining (NJ) phylogenetic trees were constructed with MEGA6 (Tamura et al., 2013). Robustness of nodes of the phylogenetic tree was assessed from 1,000 bootstrap resampling, and values > 70% were used for internal nodes.

## Results

### Assembly of the full genome of a novel badnavirus from BLRD-affected birch plants

RNA-Seq analysis was performed for five birch plants. Three of these plants (BpubFin407501_3A, BpubFin407507_3I and BpenGer407526_5M) were BLRD-affected symptomatic young seedlings that had been graft-inoculated from infected sources of Finnish or German origin (Table 1). The two other plants were symptomless controls (BpubGerNo4 and BpendGerM0197542) from Germany. For the three BLRD-affected plants, BLASTN and BLASTX annotation of the assembled contigs revealed from one to seven large contigs exhibiting high BLAST scores with members of the badnavirus genus (Table 1). In addition, plant BpenGer407526_5M—already demonstrated to be infected by CLRV—yielded ten CLRV contigs, with a cumulative length nearly equivalent to that of the CLRV genome (Table 1). Regarding the two symptomless control plants, no contigs with homology to either CLRV or badnaviruses were recovered (Table 1).

Ultimately, contigs could be assembled into scaffolds covering near complete badnaviral genomes for plants BpenGer407526_5M and BpubFin407501_3A. In the case of plant BpubFin407507_3I, the single contig obtained already represented a nearly complete genome. Overall, the *de novo* assembly process allowed the reconstruction of long contiguous near complete badnaviral genome sequences of 7,916 base pairs (bp) (BpubFin407501_3A), 7,973 bp (BpubFin407507_3I) and 7,846 bp (BpenGer407526_5M). In each case, deep average coverage was obtained for the scaffolds, ranging from 35.1X to 68.7X and giving excellent confidence in the assembled sequences.

PCR assays were performed using the outward-facing primer pair PolyBadna-F1/R1 to prove the circularity of the genome and to complete the missing region in the assemblies (Table 2). A 780 bp-fragment was amplified from the various plants and was sequenced. The sequences obtained perfectly matched the ends of the contigs or scaffolds assembled for the three birch plants, demonstrating the circular nature and finalizing the genomes for the three different badnavirus isolates. These genomic sequences have been deposited in the GenBank under accession numbers MG686420, MG686419 and MG686421, respectively. The variants assembled from the samples BpubFin407501_3A and BpubFin407507_3I are nearly identical, differing only at three nucleotide positions, while the variant from the sample BpenGer407526_5M diverges from them by 8.9%. According to our knowledge, this is the first discovery of a badnavirus in *Betula* species.

Further analysis of the contigs from the three BLRD plants revealed in the sample BpubFin407501_3A additional badnavirus contigs that differed from the completely assembled isolate, suggesting the presence of a second, divergent isolate in this plant. These various contigs could be assembled in a scaffold with several large gaps and having only a low 2.5X average coverage. The largest contigs integrated in this scaffold was 2,080 bp-long and the scaffold showed about 8.3%—10.4% divergence from the fully assembled genomes. Due to the low coverage, it was not possible to complete the genome of this variant from the RNA-Seq data.

### Genome structure and sequence analysis of the novel badnavirus

**Fig 2:**
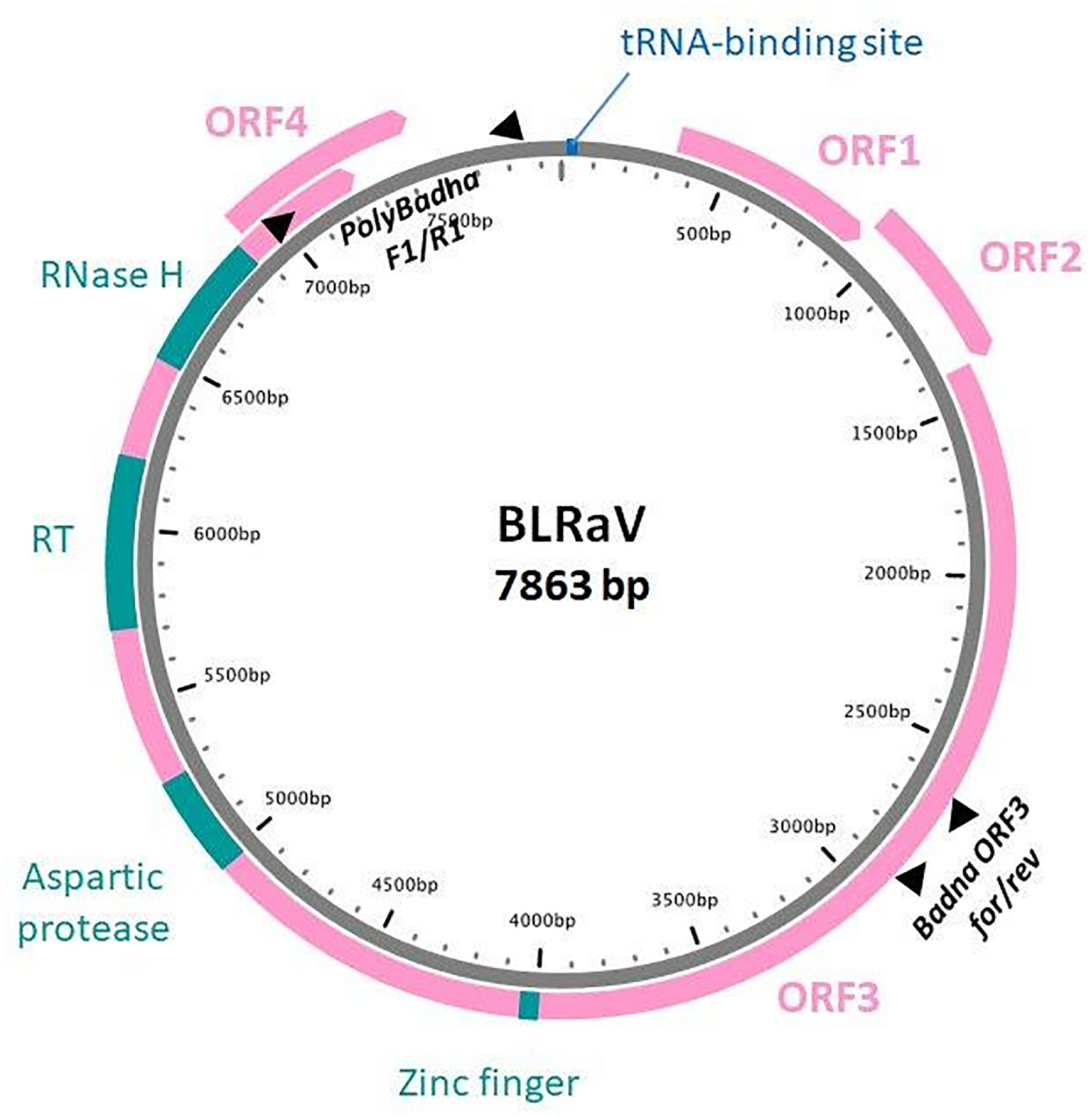
Schematic representation of the genome organization of birch leaf roll-associated virus (BLRaV). Open reading frames are shown, together with the tRNA primer binding site (defining position 1 nt on the genome) and the functional motifs identified in the ORF3 polyprotein. RT: reverse transcriptase; RNase H: ribonuclease H. The positions on the genome of the two primer pairs used in the various experiments (see Table 2) are shown by black triangles.

Depending on the isolates, the genome of the virus is 7,862–7,864 base pairs (bp), which is within the range of typical badnavirus genomes [23]. The few indels observed are in the large intergenic region, with the exception of a 3-nucleotide (nt) deletion removing one amino acid in the ORF3 protein encoded by BpenGer407526_5M. The genome shows a typical Badnavirus organization with three major open reading frames (ORFs). In the BLASTN and BLASTX analyses, ORF1 shows very low level of amino acid (aa) identity (e-value 1E-04; 27–28% aa identity) with the ORF1 protein of the badnavirus *Grapevine vein-clearing virus* [24]. ORF2 lacks a methionine initiation codon but it is possible that, similar to the ORF1 of some banana streak viruses [25], it could be translated through initiation on a non-conventional start codon. In this context, the conserved CUG codon at positions 932 nt (FIN) or 934 nt (GER) appears as a potential candidate. The encoded ORF2 protein again shows only very low level of aa identity (29%) with other badnaviral proteins, the closest being the ORF2 protein of *Grapevine Roditis leaf discoloration-associated virus* (GRLDaV, [26]). ORF3 has clear affinities with the ORF3 protein of other badnaviruses. The main conserved motifs found in other such badnaviral proteins are observed at the expected positions (Fig 2). The complete ORF3 protein of the novel virus isolates shares only from 34.6 to 44% identity with the corresponding proteins of other badnaviruses. A fourth potential ORF is observed, overlapping with the end of ORF3 (Fig 2). After pairwise comparisons of genome sequences in BLASTX, ORF4 shared very low aa identity (25%) with only one other badnaviral protein, the ORF4 protein of *Rubus yellow net virus*.

### Phylogenetic analysis of the novel badnavirus

**Fig 3:**
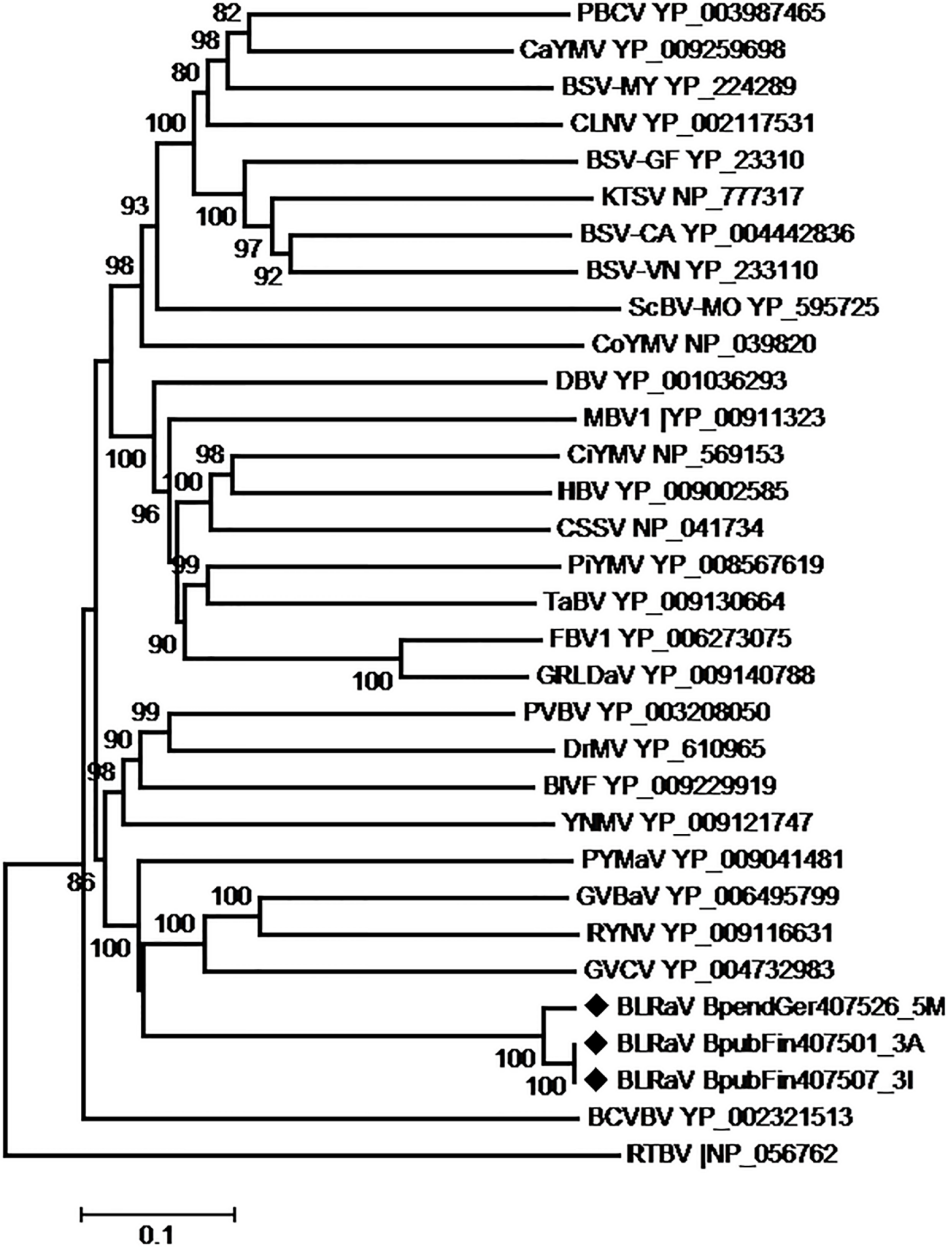
Phylogenetic tree reconstructed using the amino acid sequences of the proteins encoded by the ORF3 of badnaviruses. The tree was reconstructed using the Maximum Likelihood method and the statistical significance of branches was evaluated by bootstrap analysis (1,000 replicates). Only bootstrap values above 70% are indicated. The scale bar represents 10% amino acid divergence. The Birch leaf roll-associated virus (BLRaV) isolates sequenced during the present work are marked by black diamonds. *Rice tungro bacilliform virus* was used as an outgroup. The abbreviations followed by the accession numbers are in alphabetic order: BSV-CA: *Banana streak CA virus*; BSV-GF: *Banana streak GF virus*; BSV-MY: *Banana streak MY virus*; BSV-VN: *Banana streak VN virus*; BlVF: *Blackberry virus F*; BCVBV: *Bougainvillea spectabilis chlorotic vein-banding virus*; CSSV: *Cacao swollen shoot virus*; CaYMV: *Canna yellow mottle virus*; CiYMV: *Citrus yellow mosaic virus*; CoYMV: *Commelina yellow mottle virus*; CLNV: *Cycad leaf necrosis virus*; DBV-SN: *Dioscorea bacilliform SN virus*; DrMV: *Dracaena mottle virus*; GVBaV: *Gooseberry vein banding associated virus*; GRLDaV: *Grapevine Roditis leaf discoloration-associated virus*; GVCV: *Grapevine vein-clearing virus*; FBV1: *Fig badnavirus 1*; HBV: *Hibiscus bacilliform virus*; KTSV: *Kalanchoë top-spotting virus*; MBV1: *Mulberry badnavirus 1*; PYMaV: *Pagoda yellow mosaic-associated virus*; PVBV: *Pelargonium vein banding virus*; PBCV: *Pineapple bacilliform comosus virus*; PiYMV: *Piper yellow mottle virus*; RTBV: *Rice tungro bacilliform virus*; RYNV: *Rubus yellow net virus*; ScBV-MO: *Sugarcane bacilliform MO virus*; TaBV: *Taro bacilliform virus*; YNMV: *Yacon necrotic mottle virus*.

Phylogenetic relationships between the birch badnavirus isolates and badnaviruses sequences known to date were estimated, based either on full genome nucleotide sequences comparisons or on ORF3 amino acid sequences comparisons. The topology structure was similar in both trees, irrespectively of whether the ML or NJ algorithms were used. Fig 3 shows a representative ML tree obtained using the ORF3 protein sequences. Badnaviruses are clustered in three major groups, with the novel virus isolates from birch consistently clustering within the group 3 (grouping according to Kasmi et al., [27]) and, specifically, in the same sub-cluster with *Grapevine vein-clearing virus* (GVCG), *Rubus yellow net virus* (RYNV), *Gooseberry vein banding-associated virus* (GVBaV) and *Pagoda yellow mosaic-associated virus* (PYMaV), most closely clustered to the latest. The new virus is clearly distantly related phylogenetically to all badnaviruses currently represented in the GenBank (Fig 3), which suggests that it is a new member of the genus *Badnavirus*.

Considering only the polymerase region (RT plus RNase H domains) that is typically used for taxonomic discrimination, the divergence level from the known badnavirus genomes is 27.6 to 40.6%, with *Grapevine vein-clearing virus* appearing as the closest badnavirus. According to the species demarcation criteria accepted for the genus *Badnavirus* (among them less than 80% nt identity in the polymerase domain and differences in host ranges; [23]), the badnavirus isolates identified in *Betula* species in the present work should be considered as representing a new member of the genus *Badnavirus*, for which the name *Birch leaf roll-associated virus* (BLRaV) is proposed.

### Variability of BLRaV in Germany and Finland

**Fig 4:**
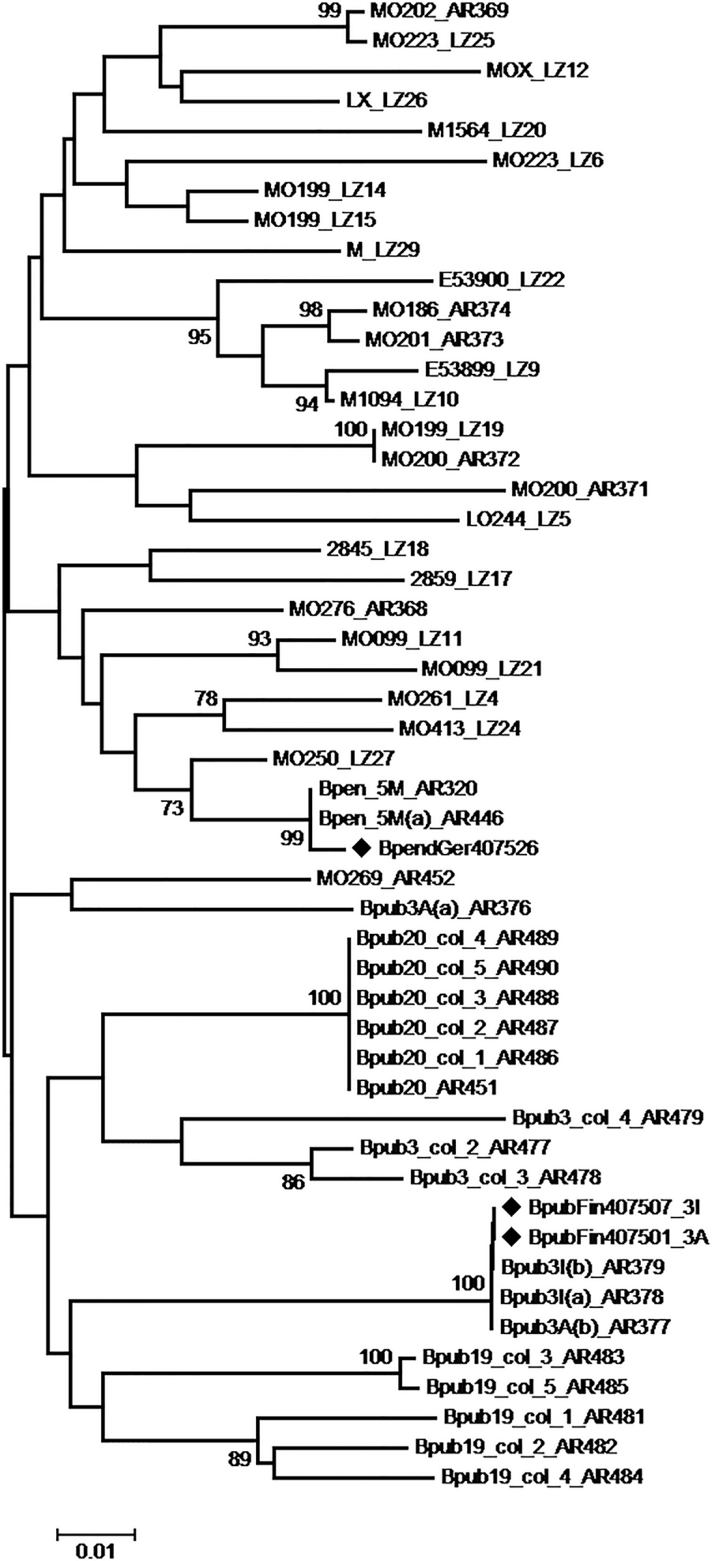
Unrooted phylogenetic tree reconstructed using the nucleotide sequences of an amplified ORF3 fragment. The tree was reconstructed using the Maximum Likelihood method and the statistical significance of branches was evaluated by bootstrap analysis (1,000 replicates). Only bootstrap values above 70% are indicated. The scale bar represents 5% nucleotide divergence. The Birch leaf roll-associated virus (BLRaV) isolates sequenced by NGS in the present work are marked by black diamonds. Sample information is provided in the S1 Table.

The presence of the novel badnavirus was confirmed by means of RT-PCR in 100% of the tested symptomatic trees, corresponding to 22 trees from six different birch stands in Berlin, and three trees located in distant urban green spots of Rovaniemi. The amplified fragment lying within the ORF3 coding region showed significant sequence variability between trees, with an average nucleotide divergence of 9.1% +/- 1% between isolates. Remarkably, in only one case did two neighbouring trees (M0199 and M0200 from Vogelsang, Berlin) share a common virus variant. On the other hand, and as expected, the ORF3 variants obtained from the birch trees used for the NGS analysis were proved identical (in the cases of the trees BpubFin407501_3A and BpubFin407507_3I) or nearly identical (in the case of the tree BpenGer407526_5M) to the sequences assembled from the NGS data (Fig 4). In the grafted seedling Bpub 3A_Fin407501, two different variants were identified: (a) Bpub3A(a)_AR376 that is phylogenetically distanced from BpubFin407501_3A and (b) Bpub3A(b)_AR377 that is identical to BpubFin407501_3A. This result is in accordance with the NGS data that indicated the presence of two variants in that plant.

All BLRaV samples from Finland and Germany used in the phylogenetic analysis cluster into a single subgroup together with the samples originating from the seedlings BpubFin407501_3A, BpubFin407507_3I and BpendGer407526, from which the full-length genome sequences were assembled in the present study. Based on the virus nucleotide variability assessed to date and the corresponding phylogenetic analysis, the existence of a single BLRaV species is suggested. Furthermore, the analysis of the phylogenetic relationships between the variants from Finland and Germany failed to provide evidence for significant clustering of isolates based on their geographical origin, despite the fact that the geographical distribution of the analysed samples within each of the sampling locations (Rovaniemi and Berlin) was very restricted (Fig 4).

It is remarkable that mixed infection by several variants was observed in several trees, first as PCR products yielding ambiguous sequences upon direct sequencing. Following cloning of PCR products and sequencing of individual clones, the presence of multiple variants in single plants was confirmed. Multiple variants occurred in Berlin trees MO099, MO199, MO200, MO223, while in Rovaniemi two trees showed contrasting patterns. In tree Bpub19 all five clones sequenced were different, but formed two distinct sub- clusters, while in tree Bpub20 all five clones sequenced revealed the same variant (Fig 4).

## Discussion

A novel badnavirus has been identified by means of RNA-Seq while investigating the etiology of a widespread disease in birch forests and urban greens throughout Europe, the “birch leaf-roll disease” (BRLD). Complete genome sequences were assembled from three birch seedlings that had been graft-inoculated with disease sources of different origins, two from Finland and one from Germany. The virus presence was confirmed in numerous trees from both countries, in different birch stands. Sequence and phylogenetic comparison of the full-genome sequences and of the ORF3 sequences of these three virus variants demonstrate that they represent a new *Badnavirus* species for which the name *Birch leaf roll-associated virus* (BLRaV) is proposed.

To date, badnaviruses are known to be distributed in tropical and temperate regions, with the majority of currently known species infecting tropical and subtropical crops such as banana, black pepper, citrus, cocoa, sugarcane, taro, and yam [20]. Each virus generally has a restricted host range, often limited to a single species. The International Committee on Taxonomy of Viruses (ICTV) listed 32 species in the genus and another 20 species are awaiting recognition by the ICTV [28]. From these, only six species have been reported in Europe; *Canna yellow mottle virus*, *Kalanchoë top-spotting virus*, *Rubus yellow net virus*, *Fig badnavirus 1*, *Grapevine Roditis leaf discoloration-associated virus* and *Gooseberry vein banding-associated virus*. The first report of a badnavirus in *Betula* species not only increases the list of hosts affected by members of this genus but also significantly expands the genus geographic distribution towards its northern limits. Given the wide distribution of *Betula* species in northern temperate and boreal climates over North America and Eurasia, the spatial distribution of this new virus species could potentially be very broad.

Similar to other pararetroviruses, badnaviruses are often also present as integrated, complete or fragmented and/or re-arranged genomic sequences in some host plant genomes and are then referred to as endogenous pararetroviruses or endogenous viral elements (EVEs) [29, 30]. The presence of EVEs is not necessarily associated with episomal gene activity. In a few cases, badnavirus EVEs are known to be able to be activated to generate viral infection (like Banana streak OL, GF or IM viruses) but in most known cases EVEs do not appear to have the potential to be activated [21]. When a new badnavirus is reported, the possibility of it being an EVE rather than an episomal virus should therefore be examined. In the case of BLRaV, convergent lines of evidence suggest an episomal nature.

First, BLRaV was efficiently transmitted by grafting to non-infected seedlings. An origin of the virus from the rootstocks rather than from the grafted scion is excluded by (i) the absence of infection in the plants grafted with non symptomatic material or in the control rootstocks themselves and (ii) the detection of identical viral variants in the grafted plants and in the original source plants.

After analysing by RNA-Seq a sample of symptomatic leaves from the original source tree (Bpub3), a 3,285 nt contig with 99.99% sequence similarity with the corresponding sequence of the full-length variant identified in the grafted seedlings (BpubFin407501_3A and BpubFin407507_3I) could be recovered (data not shown). This demonstrates that the homologies between the isolates infecting the source tree and the grafted ones extends much further than the short, 241 nt region amplified by RT-PCR in the variability analysis. These results further support the hypothesis that the only way that the grafted seedlings in Berlin could share the same BLRaV variant as the original Bpub3 tree in Rovaniemi, is by acquiring this variant through graft transmission, demonstrating the episomal gene activity of BLRaV.

Similarly, the multiple co-infections observed in both Finnish and German trees, with up to five different variants detected in a single tree, and the high BLRaV diversity (9.1% average divergence between randomly selected isolates) suggest the existence of a highly diverse and dynamic virus population with large population size (high variant diversity), effective natural transmission pathway(s) and wide geographic distribution (North Finland, Germany). These viral population features, in combination with the described emergence of the “birch leaf-roll disease” contemporaneously in various European regions [2 – 5], further confirm the hypothesis that the novel badnavirus is infectious in birches.

Even in cases where episomal replication is demonstrated, the presence of endogenous forms of the virus in the host(s) genome cannot be excluded. Some banana streak viruses (BSVs) are found in episomal form having an endogenous counterpart (eBSV), while there are other endogenous banana sequences that have no episomal counterpart [25]. Infective eBSVs constitute an extreme case of parasitism, as well as a newly described strategy for virus vertical transmission [31]. The presence of complete or re-arranged integrated version of the genome of BLRaV in the genome of *Betula* species cannot therefore be excluded.

The whole genome sequencing of dwarf birch (*Betula nana*) has been initiated in 2013 [32] and genome sequences comparison of the currently available genetic resources for that species against the newly identified BLRaV sequences using BLASTN failed to reveal evidence of integration of BLRaV. Although the *Betula* species found to host BLRaV so far (*B*. *pubescens* and *B*. *pendula*) are genetically different from *B*. *nana*, this observation would seem to rule out ancestral integration events prior to the separation between these species.

The discovery of BLRaV in birches exhibiting symptoms of BLRD drastically changes our concept on the BLRD etiology. All symptomatic trees tested were BLRaV-positive, sampled trees representing different birch stands from two distant countries. In parallel, the few asymptomatic trees tested in the frame of the present work were negative for BLRaV. These results suggest the strong association of BLRaV to the BLRD and further work is now needed to satisfy Koch’s postulates and firmly establish or rule out a causal role.

## Supporting information

S1 Table List of samples used for the BLRaV variability analysis. Samples correspond to partial ORF3 sequences and represent BLRaV variants detected in symptomatic trees in Berlin (Germany) and Rovaniemi (Finland).
(PDF)
