# Correction: A novel *badnavirus* discovered from *Betula sp*. affected by *birch leaf-roll disease*

**DOI:** 10.1371/journal.pone.0200555

**Published:** 2018-07-06

**Authors:** The PLOS ONE Staff

In the title of this article, “badnavirus” and “birch leaf-roll disease” were incorrectly italicized. The correct title is: A novel badnavirus discovered from *Betula sp*. affected by birch leaf-roll disease. The correct citation is: Rumbou A, Candresse T, Marais A, Theil S, Langer J, Jalkanen R, et al. (2018) A novel badnavirus discovered from *Betula sp*. affected by birch leaf-roll disease. PLoS ONE 13(3): e0193888. https://doi.org/10.1371/journal.pone.0193888.

The publisher apologizes for the error.
