# Supplementary material for: A novel badnavirus discovered from Betula sp. affected by birch leaf-roll disease
Source: PLoS One. 2018 Mar 1;13(3):e0193888. doi: 10.1371/journal.pone.0193888 (PMC5833271; doi:10.1371/journal.pone.0193888)
Supplement: S1 Table — Samples correspond to partial ORF3 sequences and represent BLRaV variants detected in symptomatic trees in Berlin (Germany) and Rovaniemi (Finland). (PDF) [file pone.0193888.s001.pdf]

S1 Table

Sample	Accession number	Tree	Location
MO099_LZ11	MG686440	M0099	Vogelsang, Berlin
MO099_LZ21	MG686441		
MO186_AR374	MG686442	M0186	Vogelsang, Berlin
MO199_LZ14	MG686443	M0199	Vogelsang, Berlin
MO199_LZ15	MG686444		
MO199_LZ19	MG686445		
MO200_AR371	MG686446	M0200	Vogelsang, Berlin
MO200_372	MG686447		
MO201_AR373	MG686448	M0201	Vogelsang, Berlin
MO202_AR369	MG686449	M0202	Vogelsang, Berlin
MO223_LZ6	MG686450	M0223	Vogelsang, Berlin
MO223_LZ25	MG686451		
MO250_LZ27	MG686452	M0250	Vogelsang, Berlin
MO261_LZ4	MG686453	M0261	Pücklerstraße, Berlin
MO269_AR452	MG686454	M0269	Vogelsang, Berlin
MO276_AR368	MG686455	M0276	Vogelsang, Berlin
MOX_LZ12	MG686456	M0X	Vogelsang, Berlin
MO413_LZ24	MG686457	M0413	Vogelsang, Berlin
LO244_LZ5	MG686458	L0244	Hochsitzweg, Berlin
LOX_LZ26	MG686459	L0X	Hochsitzweg, Berlin
E53899_LZ9	MG686460	E53899	Schwarzer Grund, Berlin
E53900_LZ22	MG686461	E53900	Schwarzer Grund, Berlin
M1094_LZ10	MG686462	M1094	Argentinische Allee, Berlin
M1564_LZ20	MG686463	M1564	Argentinische Allee, Berlin
2845_LZ18	MG686464	2845	Argentinische Allee, Berlin
M_LZ29	MG686465	M	Wilskistraße, Berlin
2859_LZ17	MG686466	2859	Argentinische Allee, Berlin
Bpub 3A(a)_AR376	MG686422	Bpub 3A_ Fin407501	HU Berlin

Bpub 3A(b)_AR377	MG686423		
Bpub 3I(a)_AR378	MG686424	Bpub 3I_Fin407507	HU Berlin
Bpub 3I(b)_AR379	MG686425		
Bpen_5M_AR320	MG686467	Bpen_5M_Ger407526	HU Berlin
Bpen_5M(a)_AR446	MG686468		
Bpub3_col2_AR477	MG686426	Bpub3	Rovaniemi
Bpub3_col3_AR478	MG686427		
Bpub3_col4_AR479	MG686428		
Bpub19_col1_AR481	MG686429	Bpub19	Rovaniemi
Bpub19_col2_AR482	MG686430		
Bpub19_col3_AR483	MG686431		
Bpub19_col4_AR484	MG686432		
Bpub19_col5_AR485	MG686433		
Bpub20_AR451	MG686434	Bpub20	Rovaniemi
Bpub20_col1_AR486	MG686435		
Bpub20_col2_AR487	MG686436		
Bpub20_col3_AR488	MG686437		
Bpub20_col4_AR489	MG686438		
Bpub20_col5_AR490	MG686439		
